# Regulation of Hard Segment Cluster Structures for High‐performance Poly(urethane‐urea) Elastomers

**DOI:** 10.1002/advs.202400255

**Published:** 2024-04-11

**Authors:** Jianliang Qin, Yifei Chen, Xiwei Guo, Yi Huang, Guoqing Chen, Qi Zhang, Gaohong He, Shiping Zhu, Xuehua Ruan, He Zhu

**Affiliations:** ^1^ School of Science and Engineering The Chinese University of Hong Kong, Shenzhen Shenzhen 518172 China; ^2^ School of Chemical Engineering at Panjin Dalian University of Technology Panjin 124221 China; ^3^ State Key Laboratory of Fine Chemicals R&D Center of Membrane Science and Technology School of Chemical Engineering Dalian University of Technology Dalian 116023 China

**Keywords:** fracture energy, multiple hydrogen bonds, poly(urethane‐urea) elastomers, sustainable materials, toughness

## Abstract

Elastomers are widely used in daily life; however, the preparation of degradable and recyclable elastomers with high strength, high toughness, and excellent crack resistance remains a challenging task. In this report, a polycaprolactone‐based poly(urethane‐urea) elastomer is presented with excellent mechanical properties by optimizing the arrangement of hard segment clusters. It is found that long alkyl chains of the chain extenders lead to small and evenly distributed hard segment clusters, which is beneficial for improving mechanical properties. Together with the multiple hydrogen bond structure and stress‐induced crystallization, the obtained elastomer exhibits a high strength of 63.3 MPa, an excellent toughness of 431 MJ m^−3^ and an outstanding fracture energy of 489 kJ m^−2^, while maintaining good recyclability and degradability. It is believed that the obtained elastomer holds great promise in various application fields and it contributes to the development of a sustainable society.

## Graphical abstract

This study focuses on the effect of the arrangement of hard segment clusters on the mechanical performance of poly(urethane‐urea) elastomers. It is found that the optimized arrangement of hard segment clusters in the elastomer can not only effectively dissipate energy and increase toughness, but also avoid premature stress concentration so that the elastomer has better strength and ductility.

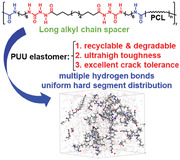

## 1 Introduction

Elastomers are materials that can undergo reversible deformation under external forces,^[^ ^1^, ^2^, ^3^, ^4^ ^]^ and are widely used in the manufacture of tires, hoses, sealing rings, and other products.^[^ ^5^, ^6^, ^7^, ^8^ ^]^ In recent years, the rapid advancement of soft robotics, wearable flexible electronic devices, and biomedical devices has become a new development direction for elastomers,^[^ ^9^, ^10^, ^11^, ^12^, ^13^, ^14^ ^]^ while at the same time, it also puts forward higher requirements for these materials, such as high strength and toughness, as well as crack‐resistance.^[^ ^15^ ^]^ Moreover, the global consumption of elastomers is huge and increases every year,^[^ ^16^ ^]^ most of which are incinerated or landfilled at the end of their service life, inevitably causing severe damage to the environment,^[^ ^17^ ^]^ and therefore, recyclability and degradability are highly desired for building a sustainable society.^[^ ^18^ ^]^ However, good mechanical properties are generally achieved through covalent crosslinking, thus limiting the recyclability and degradability of elastomers. Combining high strength and toughness, excellent crack‐resistance, degradability, and recyclability in the same material is one long‐standing challenge in the field of advanced elastomers.^[^ ^18^, ^19^, ^20^, ^21^, ^22^ ^]^

Poly(urethane) (PU) and poly(urethane‐urea) (PUU) elastomers are important members of high‐performance elastomers with highly tunable hard and soft segment microphase separation structures.^[^ ^23^, ^24^, ^25^, ^26^, ^27^, ^28^ ^]^ The hydrogen bonds between urethane/urea groups are critical for energy dissipation and therefore affect the overall performance of PU and PUU elastomers.^[^ ^29^ ^]^ For example, inspired by spider silk, Liu et al.^[^ ^30^ ^]^ designed PUU elastomers with rich hydrogen bond donors and acceptors, achieving a fracture strength of 75.6 MPa, an ultra‐high toughness of 390.2 MJ m^−3^ and an unprecedently high fracture energy of 215.2 kJ m^−2^. Their research showed that flexible alicyclic isocyanates were more suitable than rigid aromatic isocyanates for preparing PUU elastomers with high density of hydrogen bonds. Wang et al. synthesized PUU elastomers with high‐density hydrogen bonds by introducing succinic dihydrazide as a chain extender.^[^ ^31^ ^]^ Abundant hydrogen bonds were conducive to the formation of microphase separation structures and thus enhanced toughness and the obtained DPUU‐2000 elastomers had a fracture strength of 84.2 MPa, an excellent toughness of 322.8 MJ m^−3^ and a fracture energy of 68.8 kJ m^−2^. These results well demonstrated the great potential of hydrogen bonds in enhancing the mechanical properties of elastomers; however, most of these previous studies focused on introducing high‐density hydrogen bonds into the elastomer structure to enhance interactions between polymer chains and thereby provide effective energy dissipation and toughening.^[^ ^28^, ^32^, ^33^ ^]^

In this study, we explore a new research direction on the arrangement of hard segment clusters in PUU elastomers. While a strong and abundant hydrogen‐bond structure in the hard segments is necessary to dissipate energy efficiently under deformation, excessively concentrated and large hard segment clusters may result in stress concentration, leading to premature fracture of the elastomer. Therefore, it is necessary to study and rationally optimize the arrangement of hard segment clusters in the elastomer, which can not only effectively dissipate energy and increase toughness, but also avoid premature stress concentration, so that the elastomer has better strength and ductility. Herein, we select biosafe and degradable polycaprolactone (PCL) as the soft segment, ensuring the degradability of the elastomers. Moreover, PCL is easy to have stress‐induced crystallization (SIC) to resist external force damage. The hard segment consists of hydrogen‐bond‐rich groups, such as acylsemicarbazides and carbamates, spaced by flexible alkyl chains with different lengths to regulate the arrangement of the hard segment clusters in order to study its effect on the mechanical properties. As a result, compared to the PUU elastomers prepared with adipic dihydrazide (AD), the hard segment clusters were smaller and more uniformly distributed in the soft segments in the PUU elastomers using dodecanedioic dihydrazide (DD) as the chain extender, resulting in much better mechanical properties. This rational design of hard segment cluster structure shows great promise for the synthesis of next‐generation elastomers that are tough, recyclable, and degradable.

## 2 Results and Discussion

**Figure 1:**
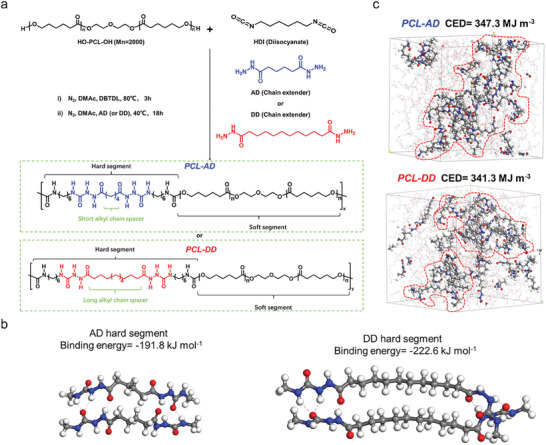
a) Synthesis of PCL‐AD and PCL‐DD. b) Optimized geometry and binding energy for two AD or DD hard segments calculated by DFT. c) MD simulations of the structures of PCL‐AD and PCL‐DD. The hard segments were bolded for clarity.

PUU elastomers were synthesized by a two‐step polymerization process in dimethylacetamide (DMAc). As shown in **Figure** **1**a, PCL diol reacted with hexamethylene diisocyanate to form an isocyanate‐terminated prepolymer, which was then extended using hydrazides to obtain the final product. In order to analyze the effect of the hard segment clusters on mechanical properties of the elastomer, we used two hydrazides having a similar structure but different carbon chain lengths, AD and DD, to extend the prepolymer. The obtained two kinds of PUU elastomers were denoted as PCL‐AD and PCL‐DD, respectively. Fourier transform infrared (FTIR) spectroscopy (Figure S1, Supporting Information), proton‐1 and carbon‐13 nuclear magnetic resonance (^1^H/^13^C‐NMR) spectroscopy (Figures S2–S5, Supporting Information) and gel permeation chromatography (GPC; Table S1, Supporting Information) were able to confirm the successful preparation of PCL‐AD and PCL‐DD.^[^ ^19^, ^34^ ^]^ The presence of hydrogen bond was evidenced by FTIR results, in which the C═O absorption bands of PCL‐AD and PCL‐DD were deconvoluted into five subpeaks, and the fraction of H‐bonded C═O in PCL‐AD and PCL‐DD were 50.1% and 49.6%, respectively (Figure S6 and Table S2, Supporting Information). The thermal properties were also studied. Dynamic mechanical analysis (DMA) showed that both elastomers had glass transition temperatures (Tg) below −50 °C and could remain in a solid state up to 180 °C (Figure S7, Supporting Information), beyond which, tan δ increased sharply, corresponding to the process of melting the elastomer. This high solid–liquid transition temperature could be attributed to the higher cohesion energy of the elastomers.^[^ ^34^ ^]^ Thermogravimetric analyses (TGA) showed that the temperature of the two elastomers at which they began to lose weight was as high as ≈250 °C (Figure S8, Supporting Information), suggesting their good thermal stability.

Density functional theory (DFT) calculations were first performed to illustrate the structures of the hard segments of PCL‐AD and PCL‐DD, respectively, as shown in Figure 1b. After structural optimization, both hard segments of PCL‐AD and PCL‐DD exhibited the formation of a maximum of six hydrogen bonds (Table S3, Supporting Information), and the binding energies between them were calculated to be as high as −191.8 and −222.6 kJ mol^−1^, respectively, indicating their strong energy dissipation capability. However, the two acylsemicarbazide groups of PCL‐DD were bent at a certain angle due to the influence of longer alkyl chains compared to PCL‐AD. This distortion in PCL‐DD was unfavorable to the aggregation of hard segments, thus inhibiting the formation of large clusters, which would ultimately affect the mechanical properties of the materials. Next, molecular dynamics (MD) simulations were conducted to estimate cohesive energy density (CED), which is the strength of intermolecular interactions per unit volume. Each amorphous cell contained three polymer chains composed of four hard segments and five soft segments, which could theoretically form up to 96 hydrogen bonds. After optimization, there were 62 and 56 hydrogen bonds in the cells of PCL‐AD and PCL‐DD, respectively, and they exhibited similar CEDs of 347.3 MJ m^−3^ (PCL‐AD) and 341.3 MJ m^−3^ (PCL‐DD). It is worth mentioning that the CEDs of these two elastomers were much higher than those of traditional rubbers (200–300 MJ m^−3^), which was mainly attributed to the strong binding energies of the hard segments evidenced by DFT calculations. These high CEDs again demonstrated that the molecular chains of both elastomers had strong intermolecular interactions, which could effectively dissipate energy and thus toughen the elastomer. More importantly, MD simulation also helped to gain insight into the structural arrangement of polymer chains prepared with different chain extenders at the microscopic level. As shown in Figure 1c, the hard segments of both PCL‐AD and PCL‐DD aggregated together among the soft segments due to the presence of abundant hydrogen bonds, forming the microphase separation structure. However, the hard segments of PCL‐DD were more evenly distributed among the soft segments rather than densely gathered together like the hard segments of PCL‐AD (Figure S9, Supporting Information), which was due to the distorted structure of the hard segments of PCL‐DD simulated by DFT and also explained why the number of hydrogen bonds in PCL‐DD was less than that in PCL‐AD. This structural difference would have a significant impact on the mechanical properties of the resulting elastomers.

**Figure 2:**
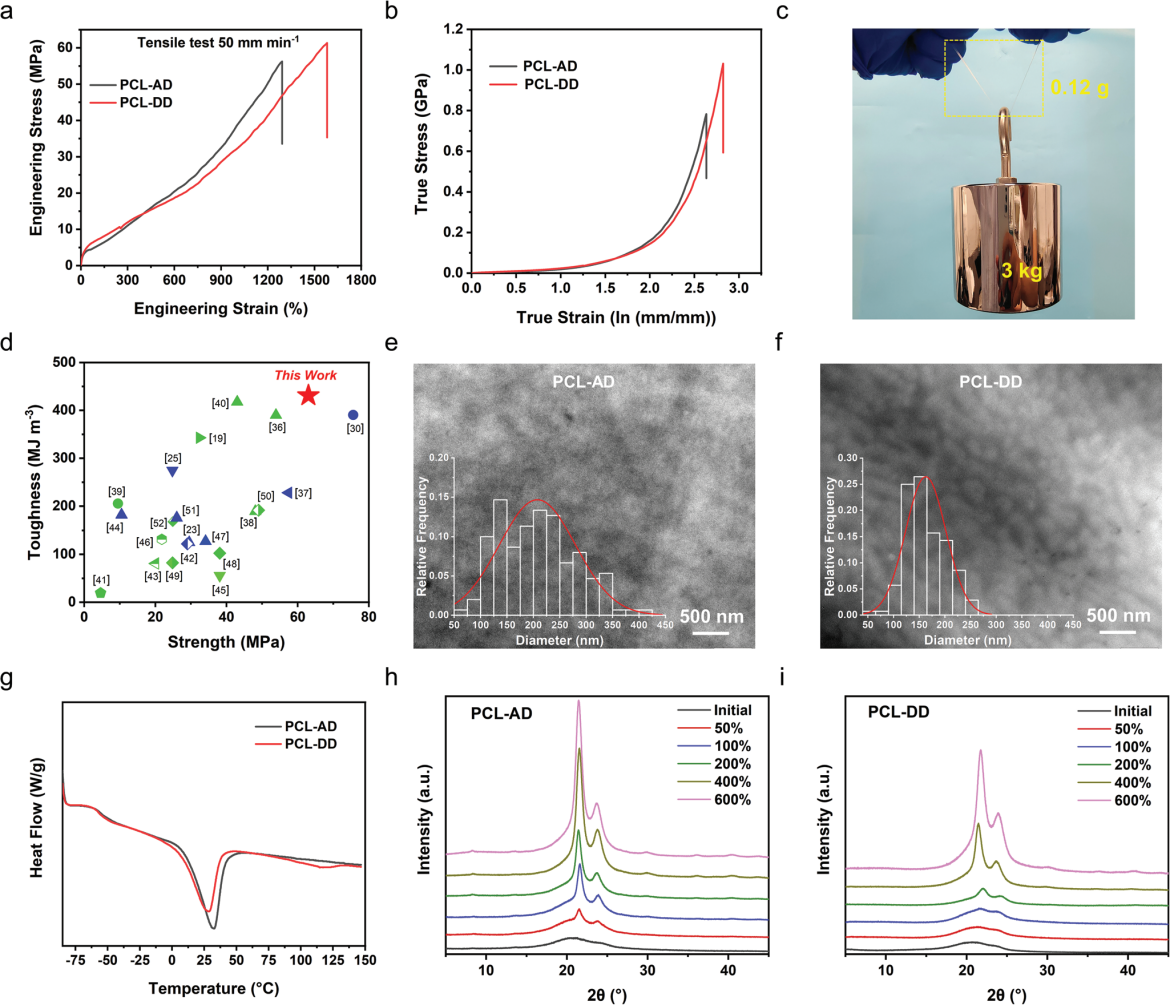
a) Engineering stress–strain curves of the PCL‐AD and PCL‐DD elastomers, measured at the strain rate of 50 mm min^−1^. b) True stress–strain curves of the PCL‐AD and PCL‐DD elastomers. c) Photograph of a 0.12 g PCL‐DD elastomer lifting a weight of 3 kg. d) Comparison of the tensile strength and toughness between the PCL‐DD elastomer and some recent literature‐reported degradable and nondegradable elastomers.^[^ ^19^, ^23^, ^25^, ^30^, ^36^, ^37^, ^38^, ^39^, ^40^, ^41^, ^42^, ^43^, ^44^, ^45^, ^46^, ^47^, ^48^, ^49^, ^50^, ^51^, ^52^ ^]^ The green and blue symbols indicated degradable and non‐degradable elastomers, respectively. TEM images of the e) PCL‐AD and f) PCL‐DD elastomers. Inset: size distributions of hard segment clusters of the PCL‐AD and PCL‐DD elastomers. g) DSC curves of the PCL‐AD and PCL‐DD elastomers. WAXD results of the h) PCL‐AD and i) PCL‐DD elastomers at different strains.

Uniaxial tensile tests were then carried out at room temperature using a universal testing machine at a speed of 50 mm min^−1^, and the results were summarized in **Table** **1**. Both PCL‐AD and PCL‐DD elastomers exhibited excellent mechanical properties, as shown in **Figure** **2**a. The tensile strength of PCL‐AD was 53.9 MPa and its elongation‐at‐break was 1283%, corresponding to an extremely high toughness of 310.5 MJ m^−3^. In contrast, the tensile strength, elongation‐at‐break, and toughness of PCL‐DD were all superior to those of PCL‐AD, which were 63.3 MPa, 1511%, and 430.9 MJ m^−3^, respectively. This ultrahigh toughness of PCL‐DD was even much better than that of the toughest spider silk ever found in the world (354 MJ m^−3^).^[^ ^35^ ^]^ Figure 2b showed that the true stress at break of PCL‐DD was 1.01 GPa, comparable to that of typical spider silk (0.8–1.5 GPa),^[^ ^35^ ^]^ that is, an excellent ability of PCL‐DD to withstand external stresses under large deformation. The true stress at break of PCL‐AD (0.76 GPa) was also excellent, but slightly lower than that of PCL‐DD. Figure 2c demonstrates the excellent mechanical properties of PCL‐DD more intuitively, where a sample having 70 × 5 ×0. 31 mm in size and 0.12 g in weight was able to withstand a weight of 3 kg (>23 000 times the sample weight) without breaking (Movie S1, Supporting Information). Moreover, materials usually exhibit a trade‐off between stiffness and ductility, and it was unexpected that PCL‐DD exhibited an excellent elastic modulus of 40.0 MPa with a large elongation‐at‐break of 1511%. PCL‐AD also exhibited similar performance, with the elongation‐at‐break and elastic modulus of 1283% and 31.6 MPa, respectively. As shown in Figure 2d, the mechanical properties of PCL‐DD were outstanding among existing commercial and literature‐reported polymeric elastomers, and such mechanical properties were even rarer among degradable elastomers.

**Table 1:** Summary of the mechanical properties of the elastomers.

Sample	Ultimate engineering stress	Elongation‐at‐break	Elastic modulus	Toughness	True stress at break
	[MPa]	[%]	[MPa]	[MJ m^−3^]	[GPa]
PCL‐AD	53.9 ± 3.4	1283 ± 58	31.6 ± 1.8	310.5 ± 28.0	0.76 ± 0.07
PCL‐DD	63.3 ± 3.8	1511 ± 61	40.0 ± 3.2	430.9 ± 36.5	1.01 ± 0.09

To explain the superior mechanical properties of PCL‐DD over PCL‐AD, their original structures and the structures under deformation were investigated. Transmission electron microscopy (TEM) was used to further observe their microphase separation structures (Figure 2e,f; Figure S10, Supporting Information), in which the hard segment clusters of PCL‐AD were large and concentrated, and the hard segment clusters of PCL‐DD were small and evenly distributed in the soft segments. This result was consistent with the MD simulation, suggesting that the long alkyl chain in the hard segment of PCL‐DD could inhibit the excessive concentration of hard segments, which led to a smaller and more uniform distribution of hard segment clusters in the elastomer. On the other hand, the alkyl chain length of the extender also affected the crystallization behavior of the soft PCL segments. Broad endothermic transition peaks between 0 and 45 °C were observed in the differential scanning calorimetry (DSC) curves of both PCL‐DD and PCL‐AD, suggesting the existence of a small amount of PCL crystalline domains in the elastomers (Figure 2g).^[^ ^34^ ^]^ The endothermic peak area of PCL‐DD was smaller than that of PCL‐AD, indicating that the initial crystallinity of PCL‐DD was less than that of PCL‐AD. This was possibly due to the less aggregated and more uniformly dispersed hard segment clusters, which restricted the crystallization of the PCL segments. The wide‐angle X‐ray diffraction (WAXD) was then performed to characterize the structural changes of PCL‐AD and PCL‐DD under deformation. Initial WAXD patterns of both elastomers exhibited a broad diffraction peak centered at 2θ = 20.8° and a shoulder peak at 2θ = 23.5° (Figure S11, Supporting Information), corresponding to the amorphous PCL segments and (200) plane of PCL crystals, respectively. In the WAXD pattern of PCL‐AD, strong diffraction peaks emerged at 2θ = 21.5° and 2θ = 23.9° at small strains (50%), corresponding to the (110) and (200) planes of the PCL,^[^ ^19^ ^]^ respectively, and became sharper with increasing strain (Figure 2h), while the broad peak of the amorphous structure gradually disappeared. The appearance of crystallization peaks indicated that the SIC of PCL chains in the elastomer occurred under the external force. In contrast, the amorphous peak was still observed at 50%, 100%, and 200% strain in the WAXD pattern of PCL‐DD, whereas the peaks corresponding to the PCL crystal only started to become evident at 200% strain (Figure 2i). This again suggested that the crystallization behavior of PCL was limited owing to the less aggregated and more uniformly distributed clusters of hard segments. Combining the differences in the mechanical properties between PCL‐AD and PCL‐DD, it became clear that large and concentrated hard segment clusters, as well as early occurrence of SIC could cause stress concentration and premature fracture of the elastomer. Therefore, rational design of the arrangement of hard segment clusters is of great importance in synthesizing high‐performance materials.

**Figure 3:**
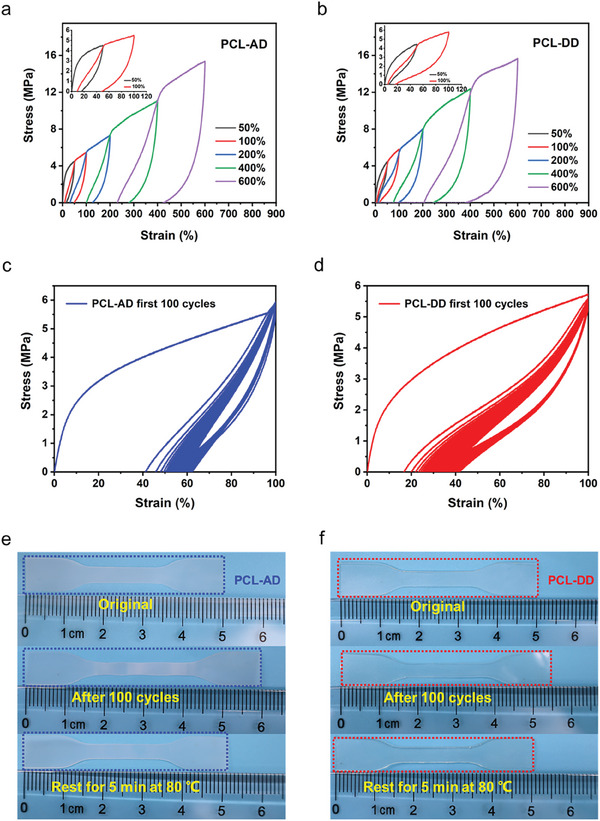
Cyclic loading–unloading tensile curve of the a) PCL‐AD and b) PCL‐DD elastomers in successive cycles with increasing strain but no resting time. Cyclic loading–unloading tensile curves of the c) PCL‐AD and d) PCL‐DD elastomers subjected to the first set of 100 cycles at a strain of 100% under a deformation rate of 50 mm min^−1^. Photographs of the original, elongated, and recovered e) PCL‐AD and f) PCL‐DD elastomers.

Cyclic tensile tests were also performed to study the effect of hard segment cluster arrangement on the elastic restorability and fatigue durability of the synthesized elastomers. Successive loading–unloading cycles with increasing strain were first conducted (**Figure** **3**a,b). Due to the negligible SIC of PCL‐DD at small strains of 50% and 100% (Figure 2i), its residual strains were ≈7% and 20%, respectively, which were much lower than those of PCL‐AD (≈16% and 43%, respectively). When the strains were larger than 200%, obvious SIC occurred in both PCL‐AD and PCL‐DD, and thus the residual strains of both elastomers were large, but the residual strains of PCL‐DD were still slightly smaller than those of PCL‐AD. Next, cyclic stretching was performed at 100% strain. The loading‐unloading tensile curves of PCL‐AD exhibited an obvious hysteresis loop (hysteresis ratio of 79.6% and dissipation energy of 3.2 MJ m^−3^) in the first stretch‐rebound cycle (Figure 3c). PCL‐DD exhibited a similar phenomenon with a smaller hysteresis ratio of 70.3% and dissipation energy of 2.8 MJ m^−3^ in the first stretch‐rebound cycle (Figure 3d). The high dissipation energies were mainly due to the large CEDs of both elastomers. After 100 cycles of stretching, PCL‐AD and PCL‐DD exhibited ≈60% and 40% residual strains, respectively. The lower residual strain of PCL‐DD could be attributed to its delayed occurrence of SIC. It was also worth emphasizing that for both PCL‐AD and PCL‐DD, the curves corresponding to the 50th‐100th cycles almost coincided with each other (Figure S12, Supporting Information), indicating that both elastomers had excellent fatigue resistance performance. After heating at 80 °C for 5 min, the PCL‐AD and PCL‐DD elastomers subjected to 100 cycles of stretching showed good elastic recovery, and the residual strains were very small, ≈8% and 3% for PCL‐AD and PCL‐DD, respectively (Figure 3e,f). The better elastic recovery of PCL‐DD could be attributed to its smaller hard segment clusters. A further cyclic tensile test was performed on the recovered sample and stress softening (Mullins effect) was observed (Figure S13). This was because stretching induced the reconfiguration of polymer chains and thus a relatively low stress was required for the second round of the tensile test.

**Figure 4:**
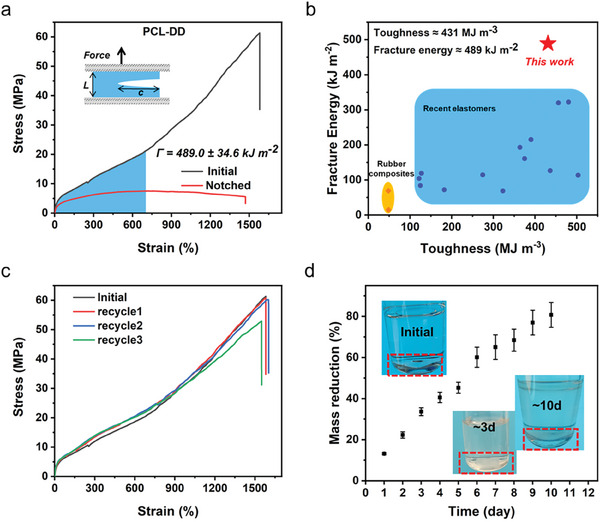
a) Tensile curves of intact and notched PCL‐DD elastomer. b) Fracture energy versus toughness of rubber composites^[^ ^53^, ^54^ ^]^ and recent elastomers.^[^ ^18^, ^19^, ^23^, ^25^, ^28^, ^30^, ^31^, ^34^, ^42^, ^44^, ^47^, ^55^, ^56^, ^57^, ^58^ ^]^ c) Tensile curves of intact and recycled PCL‐DD elastomer. d) Degradation of PCL‐DD elastomer in lipase PBS solution, inset: photographs of the degradation process of PCL‐DD elastomer.

Thanks to the existence of abundant hydrogen bonds and SIC of PCL, the obtained elastomers also demonstrated excellent crack resistance, which was evaluated by the fracture energy (Г). As shown in **Figure** **4**a, the fracture energy of PCL‐DD measured by the pure shear method was 489.5 kJ m^−2^, while that measured by the trouser tear test was 531.1 kJ m^−2^ (Figure S14 and Movie S2, Supporting Information). The results of the two methods were generally consistent. On the other hand, the fracture energies of PCL‐AD measured by these two methods were slightly lower, 358.9 and 404.7 kJ m^−2^, respectively (Figure S15, Supporting Information). Notably, the ultrahigh fracture energy of PCL‐DD was very rare in elastic materials, which was more than 48 times that of natural rubber (ca. 10 kJ m^−2^). This higher fracture energy enabled the notched sample to lift a weight of ≈18 000 times its mass without rupturing (Figure S16 and Movie S3, Supporting Information), which ensured that the elastomers could maintain a long service life. The toughness and fracture energy of PCL‐DD and various materials were summarized in Figure 4b, which clearly demonstrated the excellent performance of PCL‐DD.

Finally, the recyclability and degradability of PCL‐DD were determined. First, due to the absence of permanent covalent cross‐linking in the polymer matrix, PCL‐DD could be conveniently recycled using solvent. As shown in Figure S17, PCL‐DD fragments were dissolved in DMF and then re‐molded into a complete film. The stress‐strain curves of the recycled PCL‐DD elastomer almost overlapped with that of the pristine one even after three cycles of recycling (Figure 4c), confirming the excellent recyclability of the PCL‐DD elastomer. Thereafter, the degradability was investigated. As the main component of the elastomer, PCL endowed PCL‐DD with excellent degradability. Herein, we tested the degradability of PCL‐DD with lipase in phosphate‐buffered saline (PBS). The initial solution was colorless and transparent, but after 3 days of enzymatic degradation, the solution became cloudy and the surface of the PCL‐DD film became very rough (Figure 4d). The mass loss of the elastomer samples was recorded at different degradation times, and ≈80% of the samples completed degradation within 10 days. Scanning electron microscopy (SEM) further visually confirmed the degradability of PCL‐DD (Figure S18, Supporting Information). The surface of the initial film sample was very smooth, but after 3 days the surface became very rough with obvious cracks, and the cracks propagated through the entire film after 10 days. At the same time, PCL‐AD exhibited similar recycling and degradation properties (Figure S19 and S20, Supporting Information).

## 3 Conclusion

In summary, we have synthesized and compared two poly(urethane‐urea) elastomers with similar composition but different chain microstructures. By tuning the alkyl chain length of the chain extender, the arrangement of the hard segment clusters in the elastomers was regulated. The hard segment clusters of PCL‐DD prepared with longer alkyl chains were more evenly distributed and had smaller sizes than those of PCL‐AD prepared with shorter alkyl chains, which were larger and more concentrated. As a result, the obtained PCL‐DD elastomer exhibited improved mechanical performance, having a fracture strength of 63.3 MPa, true stress at break of 1.21 GPa, an ultrahigh toughness of 431 MJ m^−3^, and outstanding crack resistance with a fracture energy of 489 kJ m^−2^. In addition, PCL‐DD had good recyclability due to the absence of permanent cross‐linking, which could be reprocessed multiple times by using solvent without affecting the mechanical properties. The introduction of PCL as the main component also enabled PCL‐DD to have good degradability. As a reliable and durable material, PCL‐DD elastomer with many advantages is expected to show great promise in fields such as defense industry, biomedicine, and flexible electronics.

## 4 Experimental Section

Detailed Experimental Section can be found in the Supporting Information.

## Conflict of Interest

The authors declare no conflict of interest.

## Author Contributions

J.Q. and H.Z. conceived the idea, designed the research, analyzed the results, and drafted the manuscript. Y.C. and X.R. performed the simulation. X.G., Y.H., and G.C. helped with the characterization. S.Z., G.H., H.Z., X.R., and Q.Z. supervised the project.

## Supporting information

Supporting Information

Supplemental Movie 1

Supplemental Movie 2

Supplemental Movie 3

## Data Availability

The data that support the findings of this study are available from the corresponding author upon reasonable request.
