# Supplementary material for: Regulation of Hard Segment Cluster Structures for High‐performance Poly(urethane‐urea) Elastomers
Source: Adv Sci (Weinh). 2024 Apr 11;11(22):2400255. doi: 10.1002/advs.202400255 (PMC11165464; doi:10.1002/advs.202400255)
Supplement: Supplementary file 1 — Supporting Information [file ADVS-11-2400255-s004.pdf]

ADVANCED SCIENCE
Open Access

Supporting Information

for *Adv. Sci.*, DOI 10.1002/advs.202400255

Regulation of Hard Segment Cluster Structures for High-performance Poly(urethane-urea) Elastomers

Jianliang Qin, Yifei Chen, Xiwei Guo, Yi Huang, Guoqing Chen, Qi Zhang, Gaohong He, Shiping Zhu, Xuehua Ruan and He Zhu**

Supporting Information

Regulation of Hard Segment Cluster Structures for High-performance Poly(urethane-urea) Elastomers

Jianliang Qin[a], Yifei Chen[b], Xiwei Guo[a], Yi Huang[a], Guoqing Chen[a], Qi Zhang[a], Gaohong He[b,c], Shiping Zhu[a], Xuehua Ruan[b*] and He Zhu[a*]

[a] School of Science and Engineering, The Chinese University of Hong Kong, Shenzhen, Shenzhen, 518172, China

[b] School of Chemical Engineering, Dalian University of Technology, 2 Dagong Road, Panjin, LN 124221, China

[c] State Key Laboratory of Fine Chemicals, R&D Center of Membrane Science and Technology, School of Chemical Engineering, Dalian University of Technology, Dalian 116023, China

Experimental Section

1. Materials

N, N-dimethylacetamide (DMAc, 99.8%), N, N-dimethylformamide (DMF), dibutyltin dilaurate (DBTDL, 95%), hexamethylene diisocyanate (HDI), adipic dihydrazide (AD, 98%) and dodecanedioic dihydrazide (DD, 98%) were purchased from Aladdin Industrial Corporation. Lipase (100,000 u/g) was bought from Shandong Longkete Enzyme Preparation Co., Ltd. Polycaprolactone diol (PCL diol, Mn=2,000) was acquired from Guangdong Juyuan Plastic Auxiliary Chemical Co., Ltd.

2. Fabrication of the elastomer

PCL diol (12 g) was added to the three-neck round bottom flask and heated at 80 ℃ to melt. N_2 was injected into the three-neck flask to purge the air for 30 minutes to remove moist air. HDI (2.42 g) and DBTDL (0.025 g) dissolved in 3 mL DMAc were then added into the flask. The obtained mixture was further heated at 80 °C under N_2 atmosphere and stirring for 3 h and then cooled to 40 °C. Consequently, chain extender (1.05 g AD for PCL-AD and 1.55 g DD for PCL-DD) dissolved in 100 mL DMAc was added into the reaction system, which was further kept at 40 ℃ for 18 h under stirring and N_2 atmosphere. The as-obtained viscous polymer solution was next poured into homemade glass dish and then heated on a hot plate at 70 ℃ overnight to obtain the final elastomer.

3. Characterization

3.1. General characterization

Attenuated total reflectance Fourier transform infrared (ATR-FTIR) spectroscopy (Nicolet iS10, Thermo Fisher Scientific, USA), proton-1 and carbon-13 nuclear magnetic resonance (^{1}H-NMR and ^{13}C-NMR) (600 MHz JNM-ECZ600R spectrometer, JEOL, Japan) were used to determine the chemical composition of the

obtained elastomers. Differential scanning calorimetry (DSC) of samples were recorded on a TA Discovery DSC 2500 analyzer under N_2 atmosphere. The samples were heated from -80 °C to 150 °C at 20 °C min^{-1} and cooled at 20 °C min^{-1} from 150 °C to -80 °C to eliminate thermal history. Final data were collected during the second heating run from -80 °C to 150 °C at a scan rate of 10 °C min^{-1}. Wide-angle X-ray diffraction (WAXD) measurements were conducted on a Bruker D8 diffractometer using Cu Kα radiation (λ = 1.5406 Å). Thermogravimetric analysis (TGA) was conducted on a TGA 55 (TA Instruments) from 30 to 600 °C at a heating rate of 10 °C min^{-1} under air atmosphere. Dynamic mechanical analyses (DMA) were performed on the Dynamic Mechanical Analyzer Q800 (TA Instruments) under the tension mode in the temperature range from -80 to 200 °C at a heating rate of 3 °C min^{-1}. Gel permeation chromatography (GPC; Waters 1515 GPC system, Waters Corporation, US) was used to measure the molecular weight of the synthesized elastomer. Scanning electron microscope (SEM, TESCAN Mira3) was used to analyze the micromorphology of elastomer samples before and after degradation. Transmission electron microscope (TEM; Talos F200X, America) was used to observe microphase separation structure of elastomer samples. Thin slices of PCL-AD and PCL-DD elastomers were prepared by cryo-ultrathin sectioning (Leica EM UC7 & FC7, America) and stained with phosphotungstic acid before TEM tests.

3.2. Tensile tests

The mechanical properties of the samples were tested using a universal tensile machine (Instron 68TM-5, 5kN) at a speed of 50 mm min^{-1} at room temperature. Uniaxial tensile test samples were cut to a standard dumbbell shape and the size of the middle rectangular area of the sample was 4 mm wide and 17 mm long. Test length of the sample between two clamps was controlled at ~10 mm. At least five individual tensile tests were conducted for each sample.

Tensile toughness (τ) of the samples can be calculated by integrating the area under the engineering stress-strain curves, using the following equation:

$$\tau = \int_0^{\varepsilon_{max}} \sigma d\varepsilon \tag{S1}$$

where σ is the engineering stress, ε is the engineering strain, ε_{max} is the elongation-at-break of the sample.

True stress (σ_t) and true strain (ε_t) are calculated based on the engineering stress-strain curves by the following equation[1]:

$$\sigma_t = \sigma \frac{L}{L_0} = \sigma(\varepsilon + 1) \tag{S2}$$

$$\varepsilon_t = \int_{L_0}^{L} \frac{dL}{L} = ln\frac{L}{L_0} = \ln(\varepsilon + 1)$$

where σ is the engineering stress, ε is the engineering strain, L is the instant length of the deformed sample, L_0 is the original length of the sample.

For the cyclic tensile test, the loading and unloading processes of the samples at 100% strain were performed continuously for 100 times at room temperature and at a speed of 50 mm min^{-1}. Next, the samples were placed in an oven at 80°C and heated for 5 min to restore the original shape. The heated samples were loaded and unloaded again at the same rate for the second cyclic tensile test.

Fracture energy (Γ) is the energy per unit area required for crack propagation in a material and can be tested through pure-shear test and trouser tearing test.[2] For pure-shear test, the width, thickness, length between two clamps and notch size of the sample were ~25 mm, ~0.35 mm, ~5 mm and ~10 mm, respectively. The pure-shear test was performed at a speed of 50 mm min^{-1}, and the fracture energy was calculated by the following equation[2]:

$$\Gamma = W(\lambda_c) * H \tag{S3}$$

$$W(\lambda_c) = \int_0^{\lambda_c} s d\lambda$$

where H is the distance between the two clamps when the precut sample is undeformed, λ_c is the critical strain when the crack begins to propagate noticeably,

$W(\lambda_c)$ is the energy per volume of the uncut sample while stretched at the critical strain λ_c for crack propagation.

For trouser tearing test, the samples were prepared with a width of 50 mm, a length of 80 mm, a thickness of ~0.35 mm and an initial notch of 30 mm in the middle along the length direction. The trouser tearing test was conducted at room temperature at a stretching speed of 50 mm min^{-1}, and the tearing force-distance curve was obtained. Fracture energy was calculated based on the tearing force-distance curve, by the following equation[3]:

$$\Gamma = \frac{2\lambda P}{h} - 2bw$$

where P is the force on the legs of the sample, λ is the extension ratio in the legs (ratio of length of deformed to undeformed leg), h is the sample thickness, b is the width of the legs and w the strain energy density in the legs. During the test, no significant changes were observed in the legs of the samples, so λ=1 and w=0 were used.

3.3. Recycling and degradation tests

The recycling test was conducted by re-dissolving the elastomer fragments in DMF at 120 ℃. The solution was then poured into a homemade glass dish and dried overnight in a 70 ℃ oven.

For the degradation test, dried samples were weighed and placed in ~5 mL PBS (pH=7.2, Guangzhou Hewei Pharmaceutical Technology Co., Ltd.) buffer solution containing ~0.1 g lipase at room temperature. The samples were taken out every 24 hours, and the surface of the samples was cleaned with deionized water. The cleaned samples were then dried and weighed. The degradation solution was replaced every 3 days to maintain the enzyme activity. Three sets of experiments were performed simultaneously to calculate the average mass reduction rate.

4. Simulation and calculation

4.1. All-atom molecular dynamics simulations

All molecular dynamics simulations were performed through Materials Studio. The amorphous cell of the elastomer was first constructed using the Amorphous Cell module. Each cell included 3 molecular chains consisting of 4 hard segments and 5 soft segments. Then the Forcite module was used to perform geometry optimization and annealing operations on the amorphous cells with COMPASSII force field. The calculated quality was always selected as fine and the charges were assigned by the force field. Each amorphous cell experienced 10 times of gradual heating processes from 300 K to 800 K and gradual cooling processes from 800 K to 300 K under NPT ensemble at 1 atm pressure, with a total simulation time of 5 ns. Then the NVT ensemble was selected to perform a 1 ns dynamic simulation at a temperature of 298 K and a pressure of 1 atm. The Velocity Scale algorithm was used to control temperature, and the Berendsen algorithm was used to control pressure. All other parameters during the simulation were default parameters. The cohesive energy density (CED) was calculated directly using the Forcite module on the amorphous cell after completing annealing and dynamics simulations. N atoms and O atoms were selected as donors and acceptors of hydrogen bonds, respectively. The criterion for the hydrogen bond in cells was that the length of H--O bond was smaller than 2.5 Å and the angle of O--H-N was greater than 120 degrees. All hydrogen bonds were generated from the N-H structure on the hard segments, and the three polymer chains in the simulations had a total of 12 hard segments, so theoretically up to 96 hydrogen bonds could be generated. After optimization, there were 62 and 56 hydrogen bonds in the cells of PCL-AD and PCL-DD, respectively.

4.2. Density functional theory (DFT) calculations

Density functional theory (DFT) calculations were performed by Gaussian 16.[4] the B3LYP-D3(BJ) functional and 6-311 G** basis sets were used for geometry optimization of two AD hard segments or two DD hard segments. The optimized hard

segment structures were imported into Materials Studio to more clearly display the generation of hydrogen bonds. The criterion for the hydrogen bond was that the length of H--O bond was smaller than 2.5 Å and the angle of O--H-N was greater than 120 degrees.

In order to obtain the bonding energy between two hard segments, single-point energy calculations were performed at the optimized geometries using the B3LYP-D3(BJ) functional and def2-TZVP basis set. All the binding energies were corrected by the basis set superposition error (BSSE) using counterpoise command.

Supplementary Figures and Tables

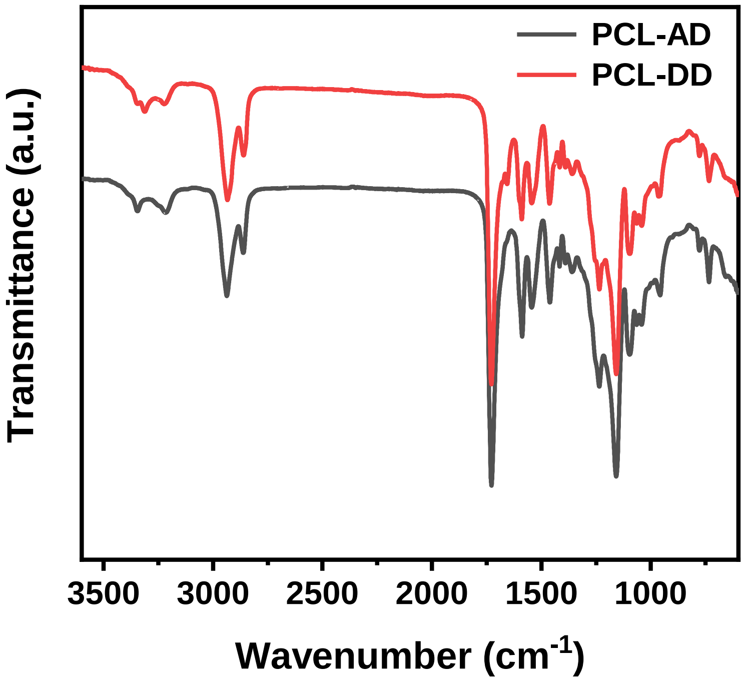

Figure S1. FTIR spectra of PCL-AD and PCL-DD elastomers.

Table S1. Summary of the molecular weights (Mn and Mw) and polydispersity index (PDI) of the PCL-AD and PCL-DD elastomers.

Sample	Mn	Mw	PDI
PCL-AD	115602	277946	2.40
PCL-DD	96727	211723	2.19

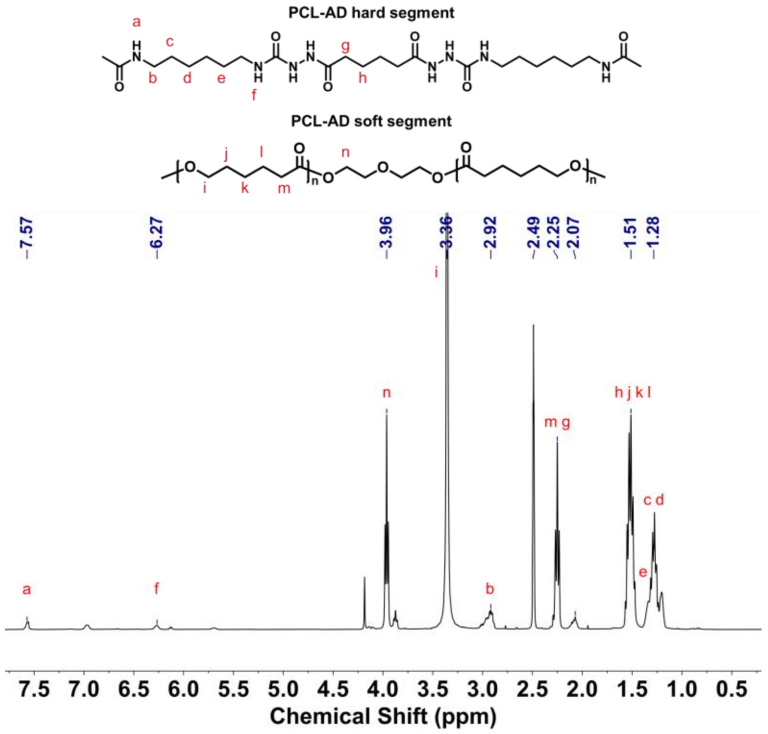

Figure S2. ^{1}H-NMR spectrum of PCL-AD in DMSO-d_6.

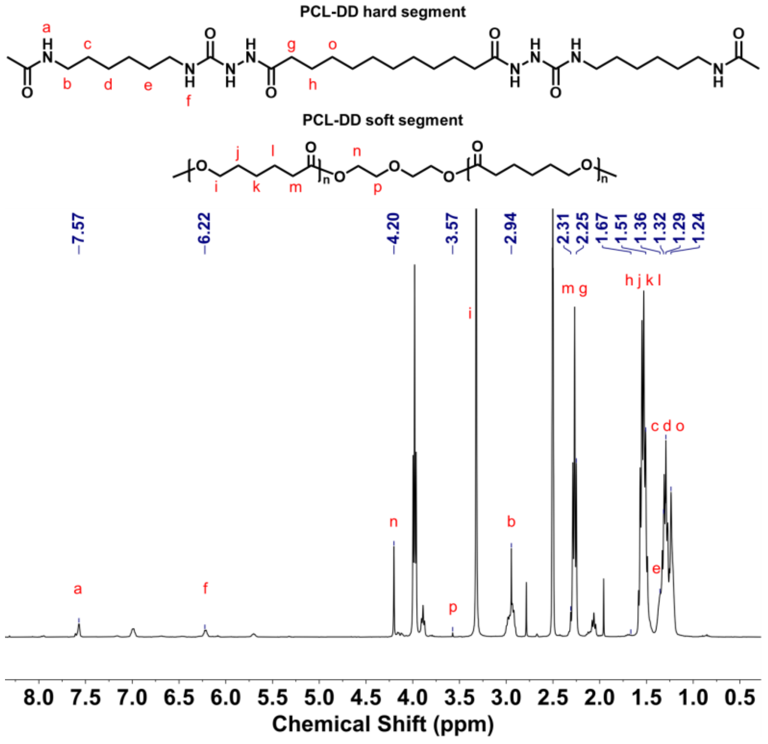

Figure S3. ^{1}H-NMR spectrum of PCL-DD in DMSO-d_6.

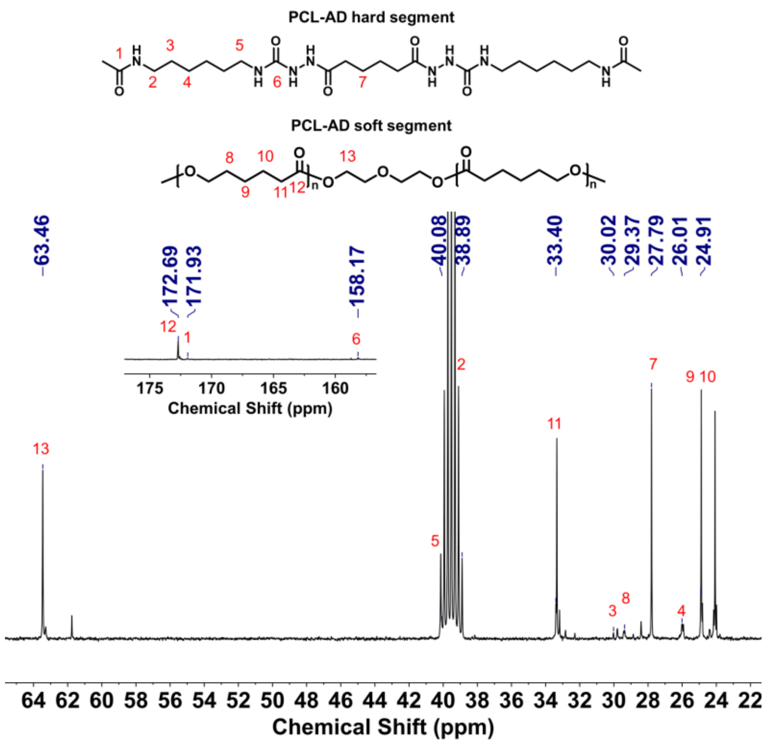

Figure S4. ^{13}C-NMR spectrum of PCL-AD in DMSO-d_6.

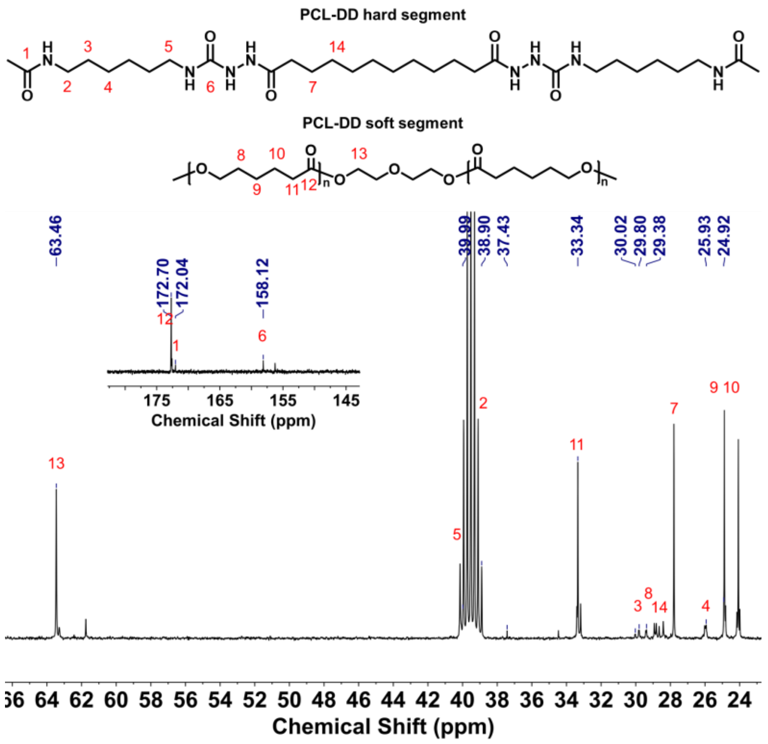

Figure S5. ^{13}C-NMR spectrum of PCL-DD in DMSO-d_6.

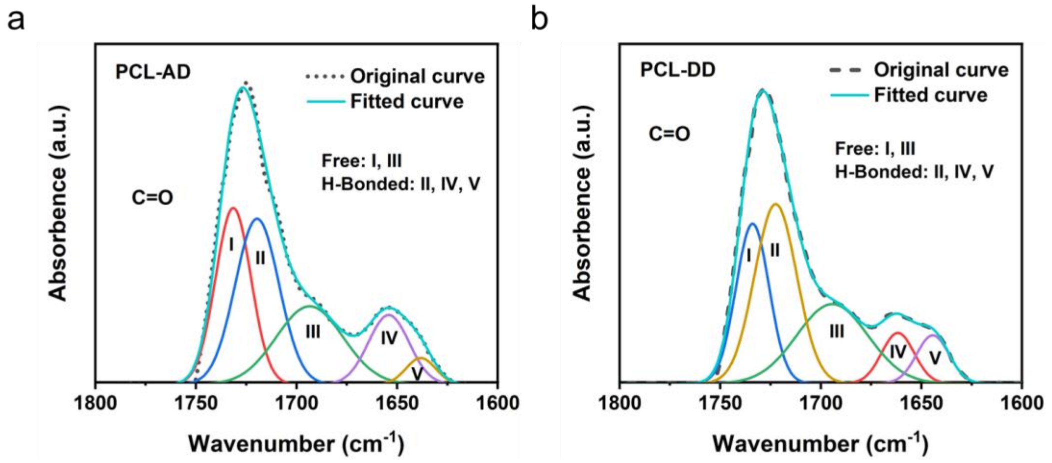

Figure S6. FTIR spectra of (a) PCL-AD and (b) PCL-DD in the C=O stretching vibration region.

Table S2. Summary of the assignment of the deconvoluted subpeaks in the FTIR C=O absorption bands for the PCL-AD and PCL-DD elastomers.

Assignment		Subpeak	Wavenumber (cm^{-1})		Area (%)	
			PCL-AD	PCL-DD	PCL-AD	PCL-DD
υ (C=O) ester urethane amide	Free	I	1731	1733	27.5	24.3
	H-bonded (Ordered)	II	1720	1722	33.0	34.8
υ (C=O) urea amide	Free	III	1693	1694	22.4	26.1
	H-bonded (Disordered)	IV	1654	1662	13.6	7.6
	H-bonded (Ordered)	V	1638	1644	3.5	7.2
Total degree of H-bonded					50.1	49.6

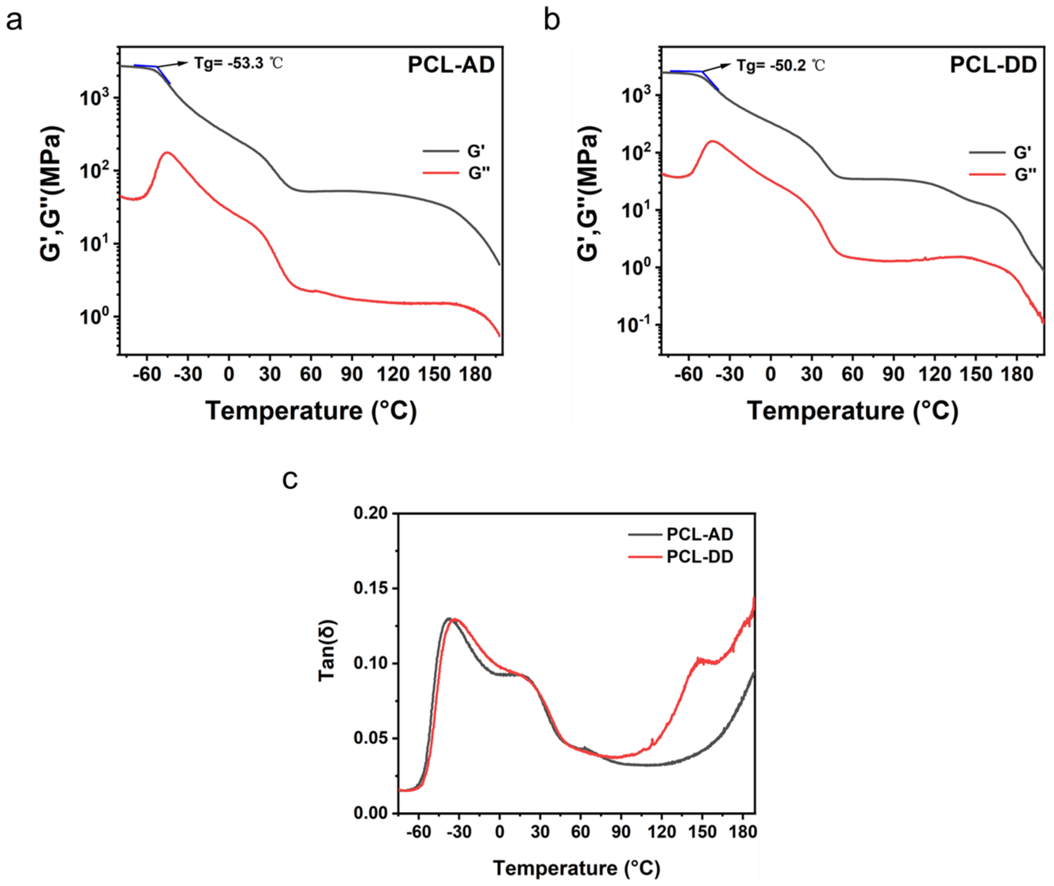

Figure S7. Temperature dependence of the storage modulus G' and loss modulus G'' of the (a) PCL-AD and (b) PCL-DD elastomers. (c) Tan δ as functions of temperature for PCL-AD and PCL-DD elastomers.

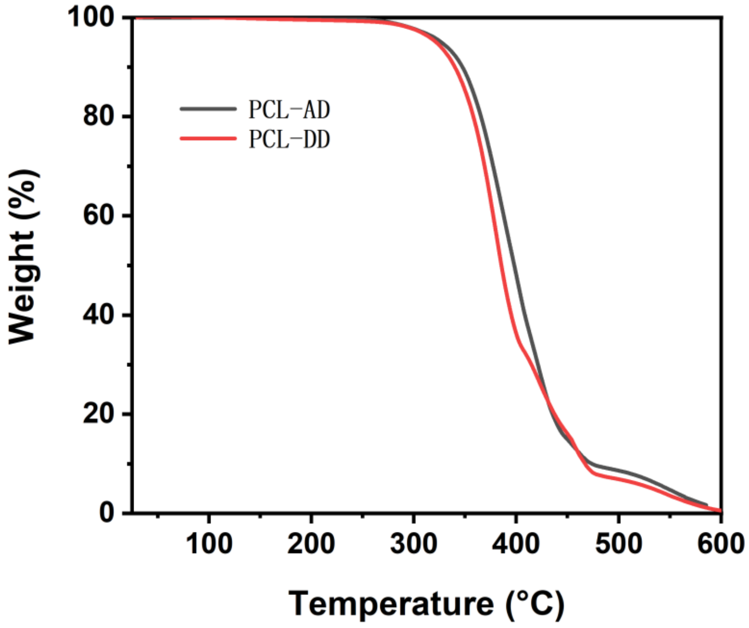

Figure S8. TGA curves of the PCL-AD and PCL-DD elastomers.

Table S3. Summary of the bond lengths and bond angles of hydrogen bonds in AD and DD hard segments.

Sample	Bond length (Å)	Bond angle (°)	Sample	Bond length (Å)	Bond angle (°)
AD hard segment	1.973	148.004	DD hard segment	2.000	149.236
	2.110	148.180		1.934	139.247
	1.777	172.043		1.674	167.609
	1.811	162.177		1.756	164.003
	2.096	145.396		2.062	148.453
	1.973	150.340		2.031	151.303
Average	1.9567	154.357	Average	1.910	153.309

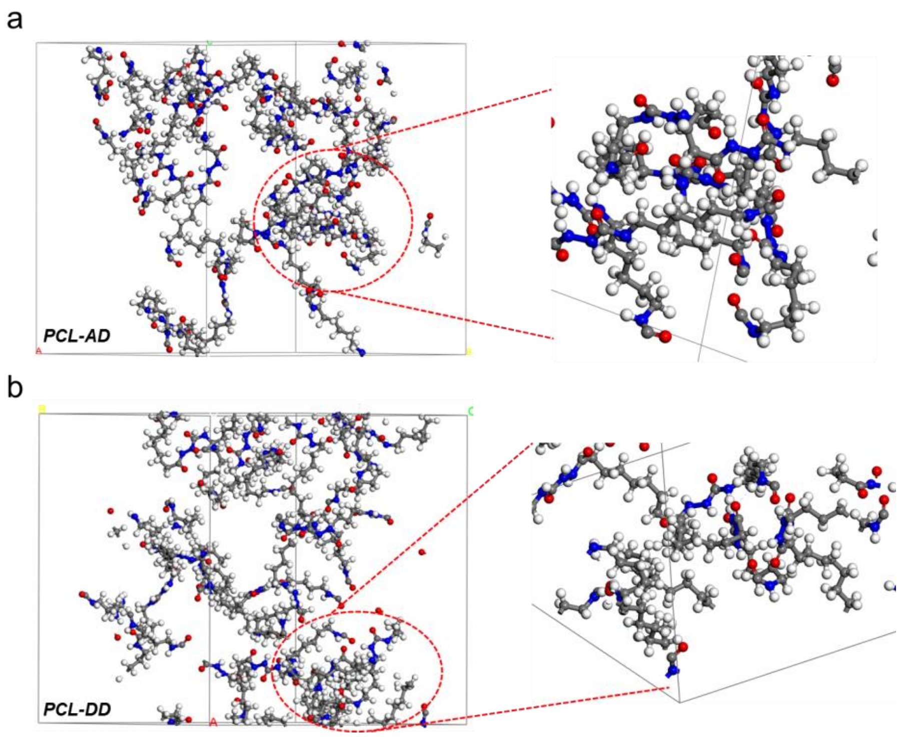

Figure S9. MD simulations of the structures of the (a) PCL-AD and (b) PCL-DD elastomers. For clearer display, parts of the soft segments were hidden. An enlarged view of the local area can be seen on the right, and the angles of these areas have been adjusted appropriately for better display.

After magnification, it could be seen that there were at most 4 hard segments clustered together in the PCL-AD cell, while at most two hard segments clustered in the PCL-DD cell. The longer alkyl chain resulted in less aggregation of the hard segments.

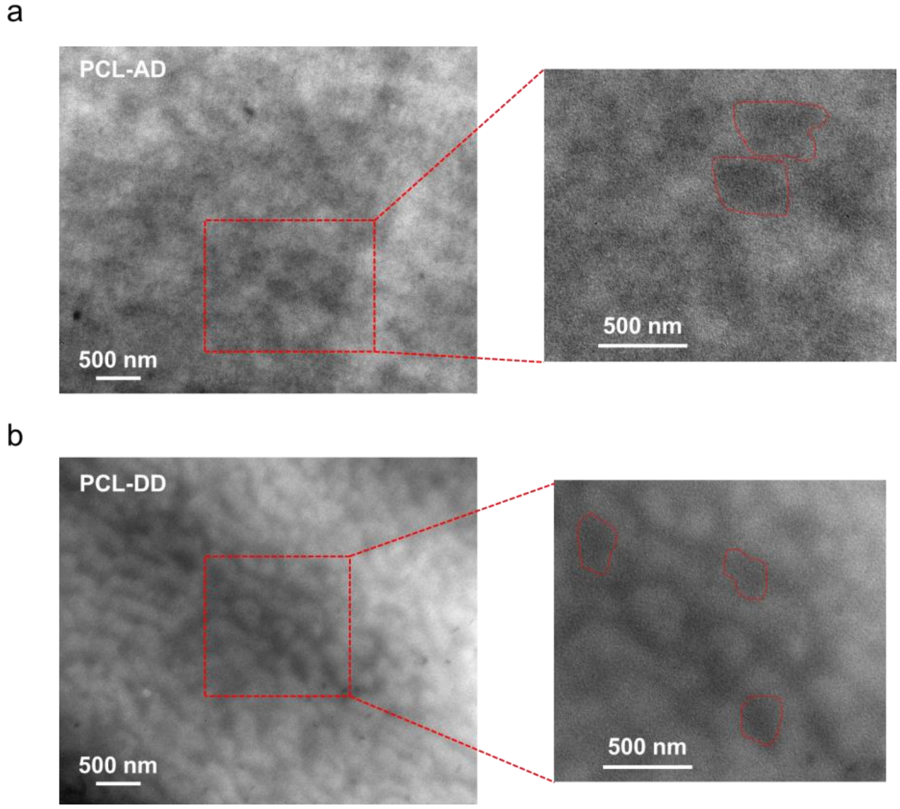

Figure S10. a, b) TEM images of the (a) PCL-AD and (b) PCL-DD elastomers.

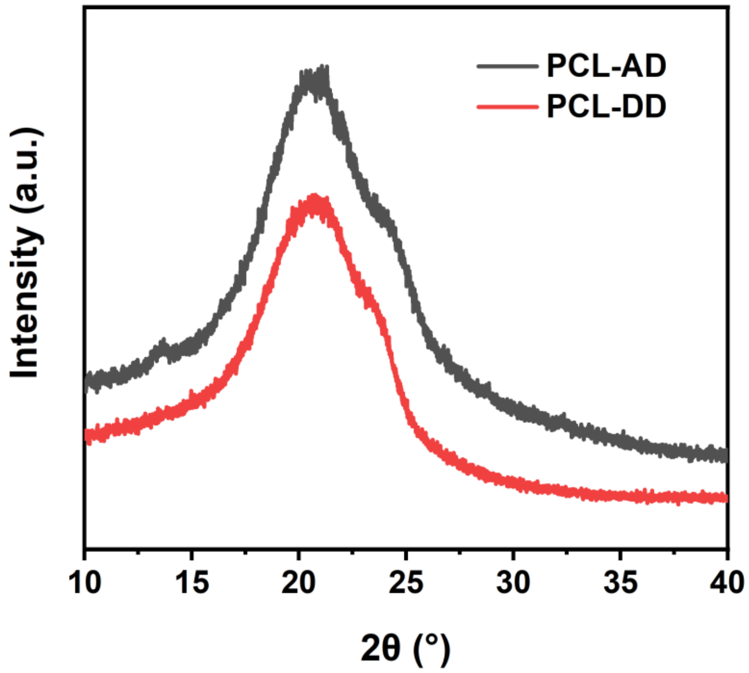

Figure S11. WAXD results of the PCL-AD and PCL-DD elastomers at initial condition.

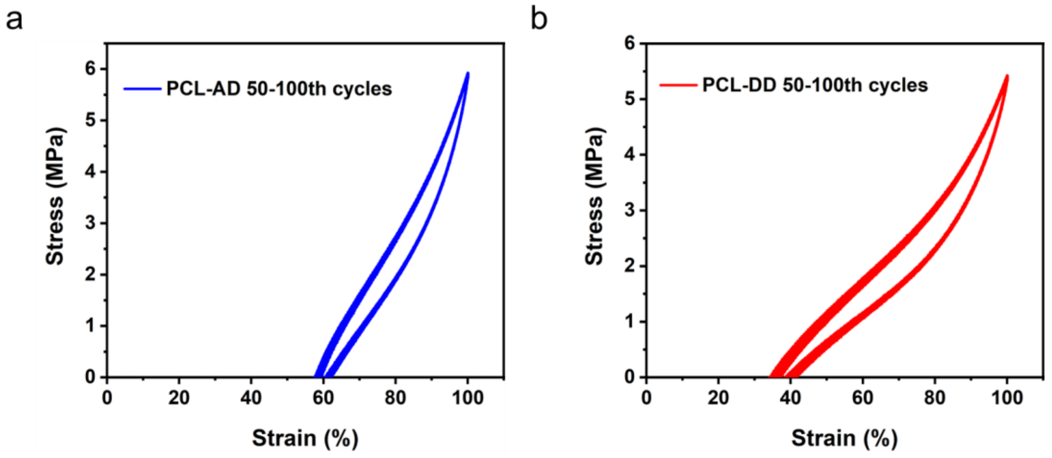

Figure S12. Cyclic tensile curves of (a) PCL-AD and (b) PCL-DD for 50–100 cycles.

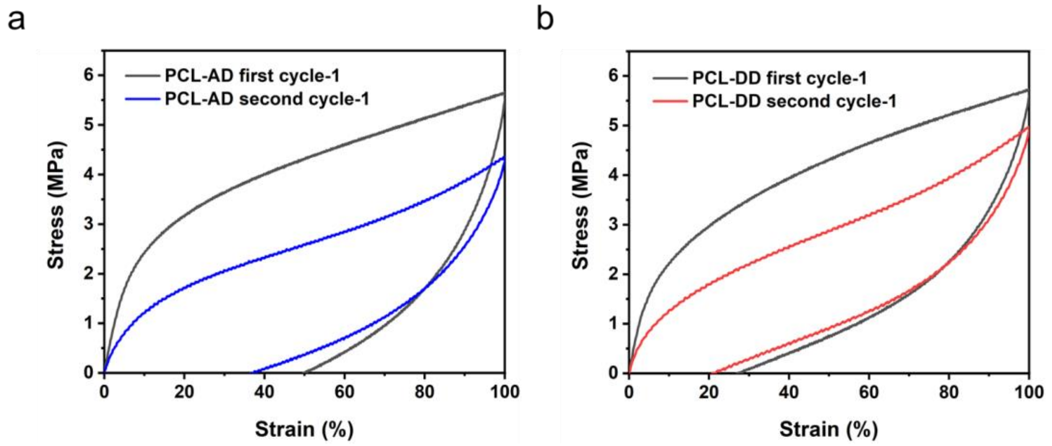

Figure S13. Cyclic loading–unloading tensile curves of the original and recovered (a) PCL-AD and (b) PCL-DD elastomers subjected to the first cycle at a strain of 100% under a deformation rate of 50 mm min^{-1}.

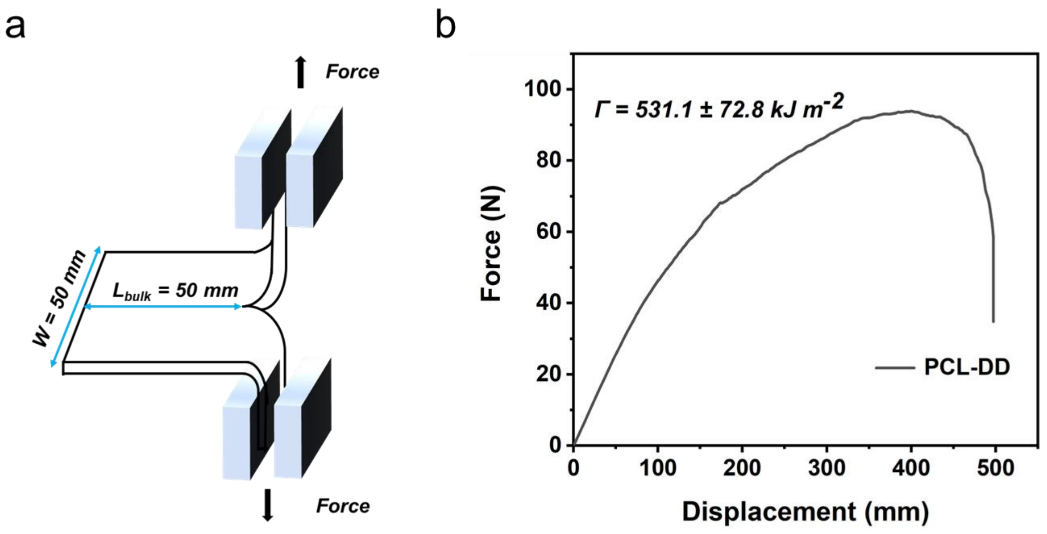

Figure S14. a) Schematic of the samples (80 mm long, 50 mm wide) for the trouser tearing test. b) Force-displacement curve of the PCL-DD elastomer (80 mm long, 50 mm wide, ≈0.42 mm thick, and 30 mm notch) measured by the trouser tearing test.

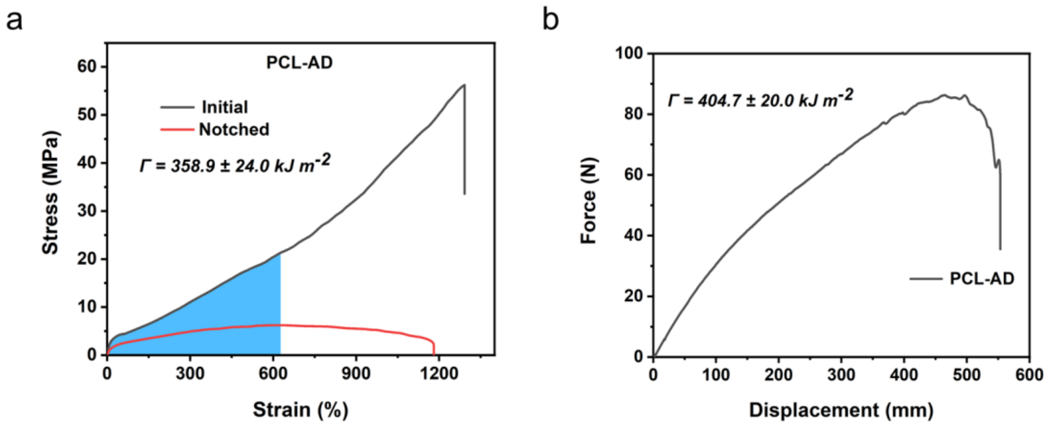

Figure S15. a) Tensile curves of intact and notched PCL-AD measured by the pure shear test. b) Force-displacement curve of the PCL-AD elastomer (80 mm long, 50 mm wide, ≈0.35 mm thick, and 30 mm notch) measured by the trouser tearing test.

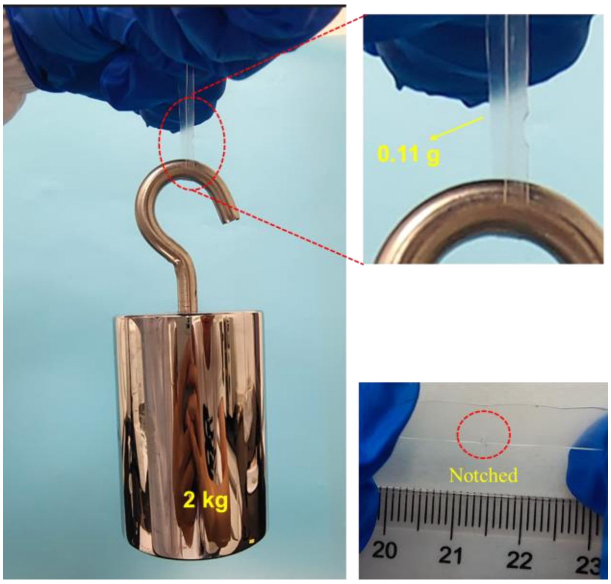

Figure S16. Photographs of ≈0.11 g notched PCL-DD elastomer lifting a 2 kg weight.

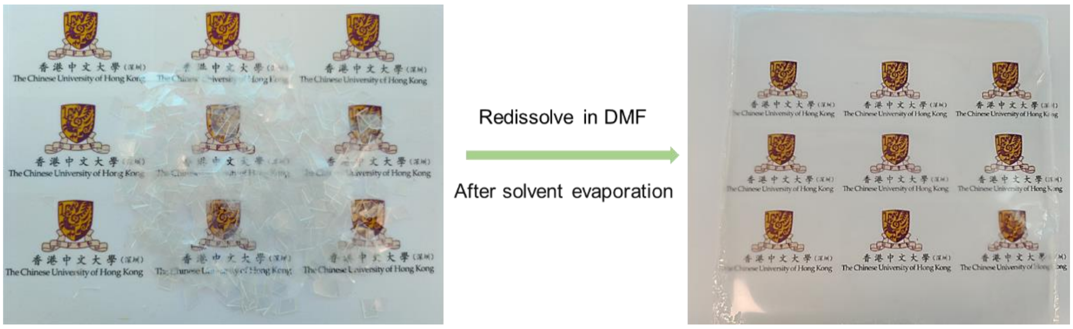

Figure S17. Solvent recycling process of the PCL-DD elastomer.

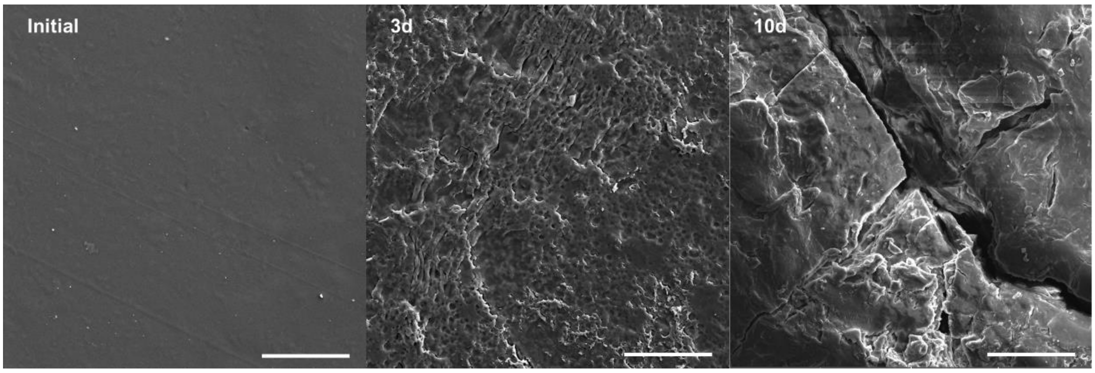

Figure S18. SEM images of the PCL-DD elastomer during degradation. Scale bar: 50 μm.

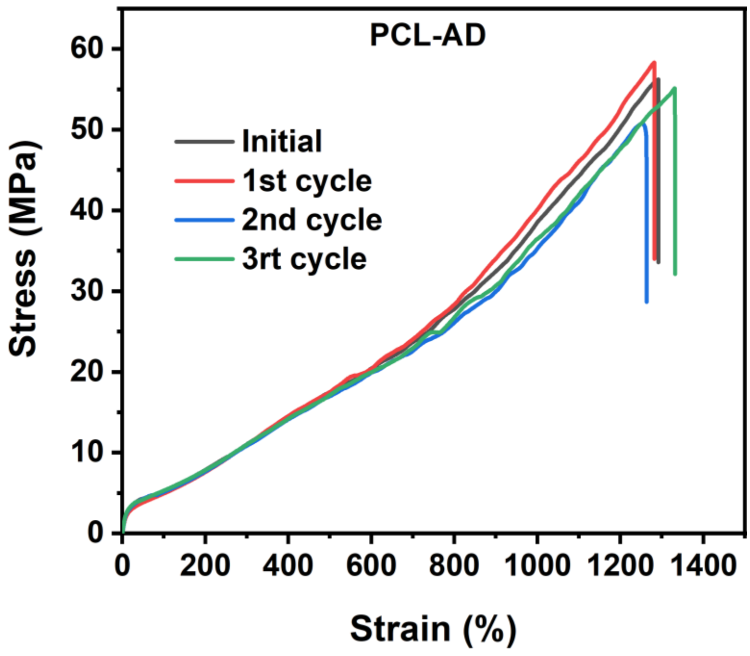

Figure S19. Tensile curves of intact and recycled PCL-AD elastomer.

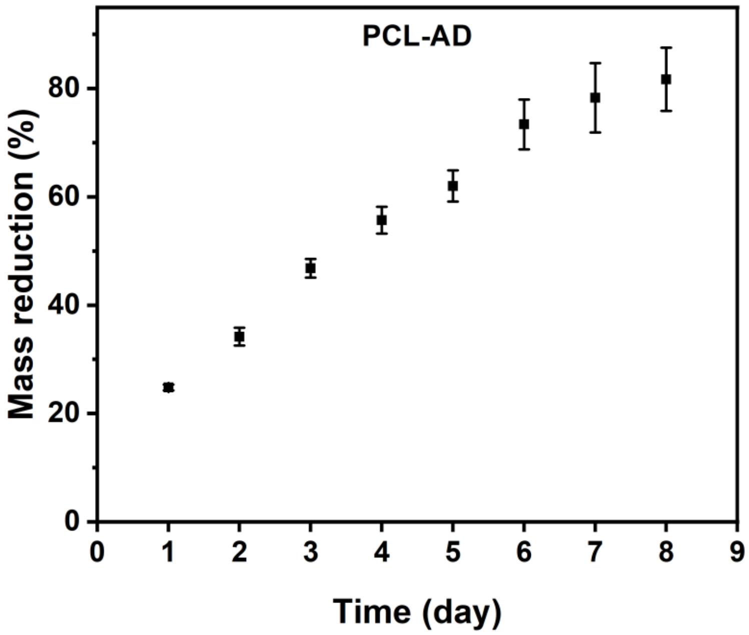

Figure S20. Degradation of PCL-AD elastomer in lipase PBS solution.

Supplementary Movies

Movie S1. Lifting a 3 kg weight by PCL-DD elastomer

Movie S2. Lifting a 2 kg weight by the pre-damaged PCL-DD elastomer

Movie S3. Elongation process of the trouser tear test for the PCL-DD elastomer

References

[1] X. Li, N. Mignard, M. Taha, F. d. r. Prochazka, J. Chen, S. Zhang, F. d. r. Becquart, *Macromolecules* **2019**, 52, 6585.

[2] W. Zhang, J. Hu, J. Tang, Z. Wang, J. Wang, T. Lu, Z. Suo, *ACS Macro Letters* **2018**, 8, 17.

[3] E. E. Gdoutos, P. M. Schubel, I. M. Daniel, *Strain* **2004**, 40, 119.

[4] M. J. Frisch, G. W. Trucks, H. B. Schlegel, G. E. Scuseria, M. A. Robb, J. R. Cheeseman, G. Scalmani, V. Barone, G. A. Petersson, H. Nakatsuji, X. Li, M. Caricato, A. V. Marenich, J. Bloino, B. G. Janesko, R. Gomperts, B. Mennucci, H. P. Hratchian, J. V. Ortiz, A. F. Izmaylov, J. L. Sonnenberg, Williams, F. Ding, F. Lipparini, F. Egidi, J. Goings, B. Peng, A. Petrone, T. Henderson, D. Ranasinghe, V. G. Zakrzewski, J. Gao, N. Rega, G. Zheng, W. Liang, M. Hada, M. Ehara, K. Toyota, R. Fukuda, J. Hasegawa, M. Ishida, T. Nakajima, Y. Honda, O. Kitao, H. Nakai, T. Vreven, K. Throssell, J. A. Montgomery Jr., J. E. Peralta, F. Ogliaro, M. J. Bearpark, J. J. Heyd, E. N. Brothers, K. N. Kudin, V. N. Staroverov, T. A. Keith, R. Kobayashi, J. Normand, K. Raghavachari, A. P. Rendell, J. C. Burant, S. S. Iyengar, J. Tomasi, M. Cossi, J. M. Millam, M. Klene, C. Adamo, R. Cammi, J. W. Ochterski, R. L. Martin, K. Morokuma, O. Farkas, J. B. Foresman, D. J. Fox, Wallingford, CT 2016.
